# Learning curve after rapid introduction of laparoscopic appendectomy: are there any risks in surgical resident participation?

**DOI:** 10.1186/s13017-016-0074-5

**Published:** 2016-05-03

**Authors:** Eszter Mán, Tibor Németh, Tibor Géczi, Zsolt Simonka, György Lázár

**Affiliations:** Department of Surgery, University of Szeged, Szőkefalvi-Nagy Béla u. 6, H-6720 Szeged, Hungary

**Keywords:** Laparoscopic surgery, Residency, Learning curve, Operative time, Complications

## Abstract

**Background:**

With the spread of the minimally invasive technique, laparoscopic appendectomy (LA) is performed with increasing frequency with excellent results. The method provides surgical residents with an excellent opportunity to learn basic laparoscopic skills and prepares them for more complex interventions.

**Methods:**

We evaluated the results of 600 laparoscopic appendectomies performed by 5 surgical residents (Group A) and 5 consultant surgeons (Group B) between 2006 and 2009. Comparing the two groups based on patient demographics, duration of surgery, operation time depending on the severity of inflammation, intraoperative blood loss, conversion rate, hospital stay in days, and postoperative complications. We also assessed the extent to which the minimum of 20 surgeries to be performed in the learning curve period as recommended by the EAES corresponds to our experience. SPPS 20 was used for the statistical analysis.

**Results:**

Six hundred laparoscopic appendectomies were performed in the study period (Group A: *n* = 319; Group B: *n* = 281). A significant difference was found between the two groups in duration of surgery during the learning curve period and when comparing the duration of LA surgeries in the learning curve period with the duration of later surgeries in both groups. The operation time in case of more severe inflammation also showed a significant difference when comparing with simple appendicitis operation time.

**Conclusions:**

The rapid introduction of laparoscopy involves few risks, the surgery is also performed with sufficient safety by surgical residents, and it provides them with an excellent opportunity to learn the basic laparoscopy skills.

## Background

In recent years, the minimally invasive technique has been used in emergency surgery in ever increasing numbers [1]. The most common urgent surgical condition to be treated with a laparoscopic method nowadays is acute appendicitis [2]. Laparoscopic appendectomy is proved to have numerous advantages over open surgery (more rapid recovery, less postoperative pain, a decrease in the need for medications and in complications from wound infections, and better cosmetic results). In addition, the procedure is reliable and safe for the treatment of this condition [3].

In many Western countries, appendectomies outside the day-shift hours are performed by surgical residents under the supervision of a consultant [4]. This is therefore the first type of laparoscopic surgery residents learn; they thus learn the basics of the minimally invasive surgical technique and may develop the basic skills they can use in later, more complex surgeries [5].

Several studies have assessed the results of laparoscopic appendectomies performed by resident surgeons (duration of surgery, hospital stay, and conversion rate) [6, 7]. It can therefore be concluded that laparoscopic appendectomy is a safe method both in the case of residents and in that of consultants. Other studies have reported that the complication rate is higher for surgeries carried out by residents [8]. Several studies have also focused on the learning curve, that is, how many surgeries are required for a surgical resident to be able to perform laparoscopic surgeries independently. These studies estimate that 20 to 30 surgeries should be performed during the learning curve [9, 10]. According to the European Association of Endoscopic Surgery (EAES) recommendation, this number is 20 [11].

At our clinic, laparoscopic appendectomy was introduced in 2006, and, over a mere six months, a complete change in approach regarding the treatment of acute appendicitis occurred, with the minimally invasive method becoming the primary approach in treating this condition. In our study, we compared resident surgeries with those performed by consultants in terms of efficacy and safety by analyzing the results of the initial, learning curve period. We also assessed the extent to which the 20 surgeries recommended by the EAES in the learning curve period correspond to our own results and experience.

## Methods

Laparoscopic appendectomy was introduced at our clinic in 2006 over a mere six months. In our retrospective study, we evaluated the results of surgeries performed by 5 residents (Group A – young resident colleagues with 2 to 3 years of surgical experience at the beginning of the study) and 5 consultants (Group B – consultant group, colleagues with 8 to 9 years of surgical experience) in the learning curve period (20 surgeries as recommended by the EAES) and in the period after that (up to Dec. 31, 2009) during routine use. Therefore, subgroups within groups A and B were created: A1 – residents, B1 – consultants in the learning curve period, A2 – residents, and B2 – consultants in the period of routine use.

*The steps for the laparoscopic appendectomy were the following:*

- *Step 1 – a pneumoperitoneum was created using a Veress needle via the umbilical access. In the case of a former abdominal operation, the umbilical trocar was introduced with the open technique (n = 27). We positioned the optical trocar in the umbilicus and two additional trocars under direct vision in the midline suprapubic area (5 mm) and left iliac fossa (10 mm).*
- *Step 2 – exploration of the abdominal cavity, isolation of the appendix. Irrigation, suction and sampling for microbiological investigation, if necessary.*
- *Step 3 – skeletisation of the mesoappendix with monopolar diathermy, clipping the appendicular artery with metal clips (two proximal clips, one distal clip).*
- *Step 4 – clipping the base of the appendix using Hem-o-lok clips (two proximal clips, one distal clip). In 8 cases, when the XL Hem-o-lok clip could not encircle the base of the appendix, we used an Endostapler (n = 6) or Endoloops (n = 2) (Group A: 2 Endostapler, 1 loop; Group B: 4 Endostapler, 1 loop). The distribution of these appendix closure methods did not differ between the groups.*
- *Step 5 – extraction of the appendix using an Endobag through a lateral 10 mm trocar.*

During emergency surgical care, the head surgeon on duty (with minimum surgical experience of 10 years) was responsible for the care at the clinic, and it was that person who decided on indication for surgery and, randomly, on the surgeon who would perform the operation. In all cases, the assistant surgeon scrubbed in, actually participated in the surgical intervention, supervised the procedure, and, naturally, gave advice to the operating surgeon, if needed, but did not “take over” the procedure.

Each resident had completed a two-week “Basic laparoscopic skills course” (training box, live animals) and had already assisted in other laparoscopic procedures (cholecystectomy, laparoscopic hernia repair, laparoscopic hiatal hernia repair, etc.). Each consultant was a more experienced laparoscopic surgeon who regularly performed other surgical procedures independently (cholecystectomy, hernia repair, etc.). Before the introduction of laparoscopic appendectomy, each surgeon was provided with theoretical training to learn the details of the technique. In both groups, the assistant was an older consultant on duty, who had the most experience in both conventional and laparoscopic procedures.

Results were evaluated for a total of 600 patients (Group A, *n* = 319 – A1: *n* = 100, A2: *n* = 219; Group B, *n* = 281 – B1: *n* = 100, B2: *n* = 181). Patient selection and data collection were performed retrospectively through an analysis of our computer database (Medsolution System) and the documentation for the patients. All patients over the age of 18 who underwent laparoscopic appendectomy in the study period were included, and none of the patients were excluded from our study.

The groups were compared based on general patient demographics (age, gender, comorbidities, and ASA score), duration of surgery, operation time depending on the severity of inflammation, intraoperative blood loss, conversion rate, hospital stay in days, negative appendectomy rate, and number of complications (early, late).

### Statistical analysis

SPSS 20 was used for the statistical analysis—the durations of surgery were compared with a two-sample *t*-test, the complications were compared with Fisher’s exact test, and the effect of inflammation on the duration of surgery was determined by analysis of variance. A significance level of *p* < 0.05 was used.

## Results

Data was evaluated for 600 patients in total between 2006 and 2009. The mean age of the patients was 38.4 years (A1: 39.6, A2: 39.3, *p* = 0.321; B1: 39.1, B2: 35.9, *p* = 0.273). Gender distribution: A1 – female: *n* = 53, male: *n* = 47; A2 – female: *n* = 119, male: *n* = 100; B1 – female: *n* = 65, male: *n* = 35; B2 – female: *n* = 98, male: *n* = 83. Regarding comorbidities (ASA score III to IV, severe cardiac disease, COPD, DM, underlying tumor disease, and chronic renal failure): A1: *n* = 10, A2: *n* = 16, *p* = 0.393; B1: *n* = 12, B2: *n* = 16, *p* = 0.281. We may thus consider these patient groups homogeneous (Table 1).

**Table 1:** Demographics by subgroup

	A1 (*n* = 100)	A2 (*n* = 219)	*p*
Gender (n)			
Female	53	119	0.283
Male	47	100	0.326
Age (years)	39.6	39.3	0.895
Comorbidities(n)	10	16	0.384
	B1 (*n* = 100)	B2 (*n* = 181)	*p*
Gender (n)			
Female	65	98	0.438
Male	35	83	0.245
Age (years)	39.1	35.9	0.263
Comorbidities(n)	12	16	0.654

A1: residents during the learning curve, A2: residents after the learning curve, B1: consultants during the learning curve, B2: consultants after the learning curve

We evaluated intraoperative blood loss in the two main groups: it was 55 mL in Group A and 45 mL in Group B, and there was no significant difference (*p* = 0.664). In Group A, conversion was required in 18 cases (5.6 %) (adhesions due to prior surgeries [*n* = 6], perforated, gangrenous appendix, the stump of which could not be treated safely with laparoscopy [*n* = 12]), while this number was 21 (7.4 %) in Group B (adhesions [*n* = 13], the stump could not be treated safely due to severe inflammation [*n* = 6], extreme obesity [*n* = 1], mesenteric injury during insufflation [*n* = 1]; *p* = 0.321). We also assessed whether the conversion rate was higher in the learning curve period: conversion was required in 14 out of 200 surgeries (7 %) in LC period subgroups A1 (residents) and B1 (consultants), while this number was 25 out of 400 (6.25 %) in routine use subgroups A2 (residents) and B2 (consultants), without a significant difference between the early and late period (*p* = 0.522). Also, there was no significant difference in hospital stay between the groups (3.21 vs. 3.84 days, *p* = 0.391, non-perforated group: Group A: 2.34 days, Group B: 2.13 days. Perforated group: Group A 4.78 days, Group B: 4.98 days). The two groups did not differ in negative appendectomy rate (NAR, 8.5 % vs. 7.8 %, *p* = 0.835) either (Table 2).

**Table 2:** Comparison of clinical datas in Groups A and B

	A (*n* = 200)	B (*n* = 400)	*p*
Blood loss (ml)	55	45	0.664
Conversion rate (n, %)	18 (5.6 %)	21 (7,4 %)	0.321
Hospital stay (days)			
Non perforated appendicitis	2.34	2.13	0.812
Perforated appendicitis	4.78	4.98	0.734
Negative appendectomy rate (NAR, %)	8.5 %	7,8 %	0.835

A: residents, B: consultants

As to duration of surgery, we evaluated whether there was a difference during the learning curve period between residents (A1) and consultants (B1), if there was a difference between the two groups after the learning curve (A2 vs. B2), and how duration of surgery changed over time in the case of residents and in that of consultants (A1 vs. A2, B1 vs. B2). We also investigated the effect of the severity of inflammation on operation time in each subgroup.

**Fig. 1:**
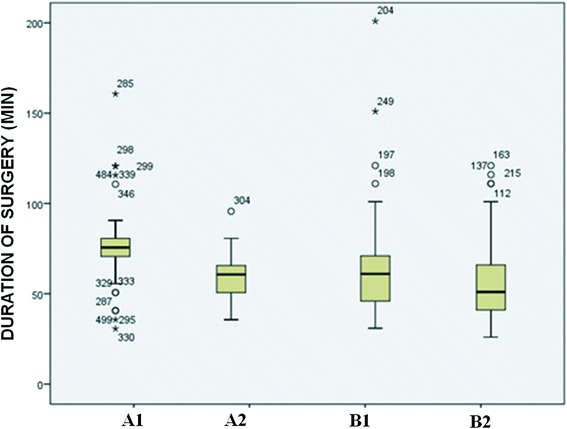
Duration of surgery by subgroup. A1: residents during the learning curve; A2: residents after the learning curve; B1: consultants during the learning curve; B2: consultants after the learning curve

The mean duration of surgery was 74.6 min in Group A1 (residents LC period), 57.3 min in Group A2 (residents routine use period), 64.13 min in Group B1 (consultants LC period) and 53.38 min in Group B2 (consultants routine use period) (Fig. 1).

When comparing the mean duration of surgery between residents and consultants in the learning curve period, a significant difference was found between the groups (A1 – residents: 74.6 min vs. B1 – consultants: 64.13 min, *p* < 0.05). The same was observed when comparing the groups after the learning curve period (A2 – residents: 57.3 min vs. B2 – consultants: 53.38 min, *p* < 0.05).

**Fig. 2:**
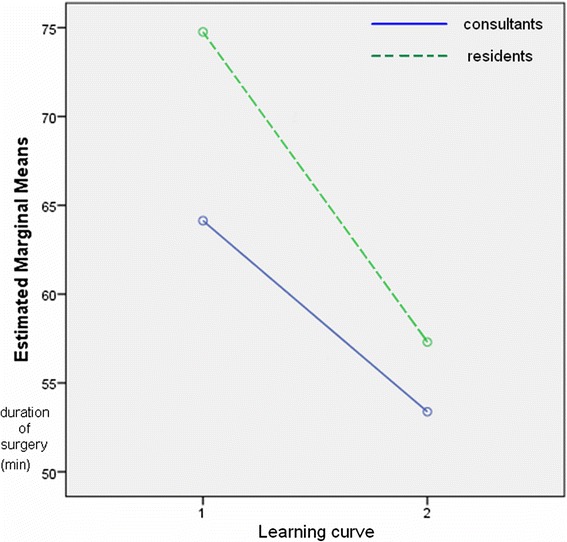
Dynamic of the duration of surgery in the learning curve period (1) and afterward (2) in the case of residents (dashed line) and in that of consultants (solid line)

In the two main groups, we compared the change in duration of surgery, the learning “dynamic”: in Group A, the duration of surgery decreased from 74.6 min to 57.3 min (*p* < 0.05), while a drop from 64.13 min to 53.38 min was observed in Group B (*p* < 0.05) (Fig. 2).

**Fig. 3:**
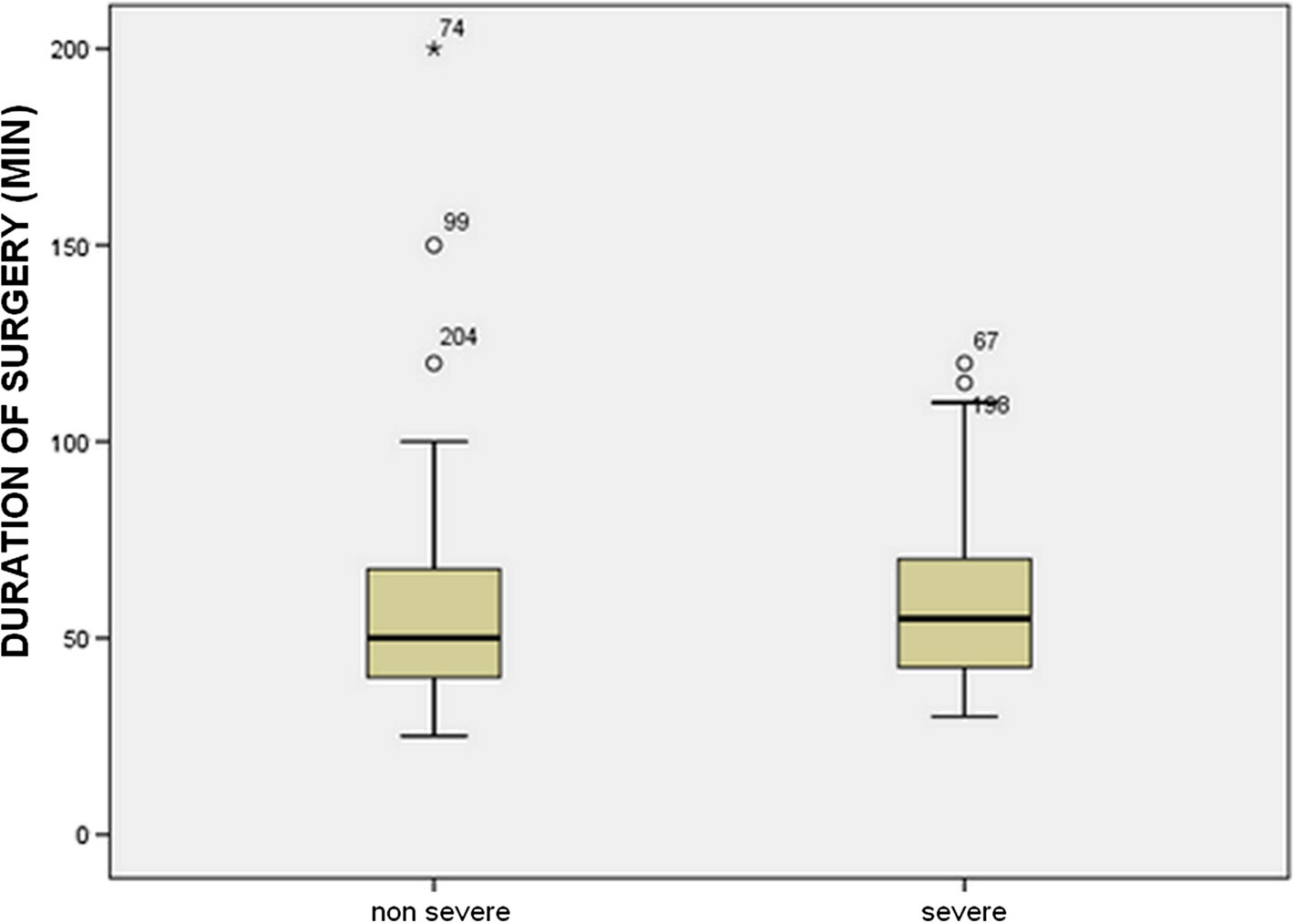
Duration of surgery depending on the severity of inflammation in Group A (residents)

When investigating the effect of the severity of inflammation on operation time, we founda significant difference between the subgroups. In Group A (residents), operation time was 61.4 min for early appendicitis with less severe inflammation (catarrhal, phlegmonous) vs. 74.8 min for severe inflammation (gangrenous, perforated) (*p* < 0.05) (Fig. 3).

**Fig. 4:**
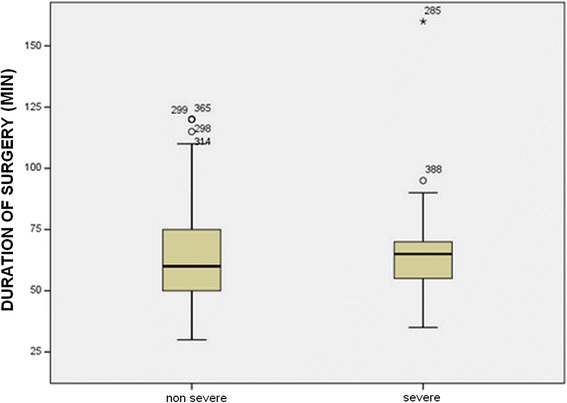
Duration of surgery depending on the severity of inflammation in Group B (consultants)

This value was 53.4 min vs. 68.5 min for Group B (consultants) (*p* < 0.05) (Fig. 4).

In the learning curve period, operation time was 58.49 min for early appendicitis and 70.12 min with severe inflammation; in the routine use period, it was 56.13 min vs. 63.34 min. We found that the severity of the inflammation affected the duration of the operation significantly when comparing Groups A and B in the LC period vs. routine use period.

The groups were also compared in terms of complications during and after the learning curve period. Early (within 30 days) major (bleeding, ileus, abscess, and thermal injury that require reoperation) and minor complications (wound infection), and late (after 30 days) complications (postoperative hernia) were assessed. There was no mortality. The types and occurrence of complications are shown in Table 3.

**Table 3:** Complications by subgroups

	A1 (*n* = 100)	A2 (*n* = 219)	B1 (*n* = 100)	B2 (*n* = 181)
Early				
Major				
Ileus	0	1	0	2
Abscess	1	1	2	1
Bleeding	1	1	2	2
Minor				
(Wound infection)	3	5	3	9
Late	–	2	2	2
Total (n,%)	5 (5 %)	10 (4.6 %)	9 (9 %)	17 (9.3 %)

A1: residents during the learning curve, A2: residents after the learning curve, B1: consultants during the learning curve, B2: consultants after the learning curve

In comparing the frequency of complications between subgroups A1 (residents) and B1 (consultants) (5 vs. 9; 5 % vs. 9 %), it can be concluded that the occurrence of complications in the learning curve period was independent of surgical experience (*p* = 0.238)

In comparing subgroups A2 (residents) and B2 (consultants) after the learning curve period (10 vs. 17; 4.5 % vs. 9.3 %), the number of complications was lower in the case of the younger group, but the difference was not statistically significant. The analysis of the same question using Fisher’s exact test did not reveal a correlation between surgical experience and number of complications.

*We used an Endostapler (n = 6) or Endoloops (n = 2) (Group A: 2 Endostapler, 1 loop; Group B: 4 Endostapler, 1 loop). The distribution of these appendix closure methods did not differ between the groups. The mean operation time in the groups was the following: Endostapler – Group A: 48.4 min; Group B: 44.2 min; Endoloops – Group A: 84.6 min; Group B: 67.3 min. We found a significant difference in the duration of surgery when comparing the Endostapler and Endoloop groups (p < 0.01). As the number of these cases were low, they did not affect the mean operation time in Group A or B significantly: Group A operation time using only Hem-o-lok clips: 65.67 min vs. using Endoloops/Endostapler as well: 65.95 min; Group B operation time using only Hem-o-lok clips: 58.325 min vs. using Endoloops/Endostapler as well: 58.755 min (Data not shown).*

## Discussion

The minimally invasive technique is used worldwide for numerous surgery types with excellent results. The open and laparoscopic techniques have been compared in numerous studies, and many advantages have been confirmed for the latter (less postoperative pain, faster recovery, lower rate of surgical infections, better cosmetic result, and less need for medication) [12–14]. It is now also generally accepted that many of the cases with severe inflammation, and even perforation, can be treated safely with laparoscopy [15, 16].

The minimally invasive technique was also introduced rapidly at our clinic, over a period of six months in 2006, and it completely superseded the open method. Considering the fact that appendicitis is an urgent surgical condition, it is treated in many cases by young resident surgeons outside the day-shift hours under the supervision of a consultant. Numerous studies have analyzed the results of laparoscopic appendectomies performed by resident surgeons. The factors evaluated were duration of surgery, hospital stay in days, complications, and conversion rate.

In our study, we also evaluated these data, comparing the results achieved by residents with the results of the surgeries performed by consultants. In addition, the results of laparoscopic appendectomies performed by the two groups were compared in the learning curve period and thereafter.

Several studies have focused on the learning curve, that is, how many laparoscopic interventions under supervision are required for a resident to be able to perform surgeries independently. The learning curve period for laparoscopic appendectomy is short; a working group has found that 2.5 procedures on average are sufficient for independent practice [17]. Other studies recommend 30 surgeries [9]. Based on the 1994 EAES recommendation, in the case of laparoscopic appendectomy, 20 surgeries are to be performed under supervision in the learning curve period for independent practice, and this is supported by several studies [5, 10, 11]. Based on our own experience, this number of surgeries is mandatory for a resident to be able to perform appendectomy independently. After the learning curve period (20 surgeries), there was a significant difference in mean duration of surgery both in the consultant group and the resident group (64.13 vs. 53.38 min and 74.6 vs. 57.3 min, respectively, *p* < 0.05). According to our results, the severity of the inflammation affected operation time significantly.

The mean hospital stay in days is a good measure of laparoscopic experience, as this period is longer in the case of a prolonged, complicated surgery. A similar objective parameter is conversion rate. In our study, there was no significant difference between the learning curve period and the period after that either in hospital stay or in conversion rate, nor was there any difference when comparing young surgeons with consultants. Conversion rate, therefore, was independent of laparoscopic experience. It was determined by the severity of the inflammation. Similarly to reports from other studies, conversion was required when the stump could not be treated safely because of the severity of the inflammation [5, 18].

Since, according to our results, there was no difference in the frequency of complications between subgroups A1 (residents) and B1 (consultants) (5 vs. 9; 5 % vs. 9 %), the occurrence of complications in the learning curve period was independent of surgical experience (*p* = 0.238). When comparing subgroups A2 (residents) and B2 (consultants) after the learning curve period (10 vs. 17; 4.5 % vs. 9.3 %), the number of complications was lower in the case of the younger group; however, this drop was not statistically significant. In a recent multicenter US study, the data for 54,467 appendectomies performed between 2005 and 2009 was analyzed. It was found that the duration of surgery is significantly longer and the number of major postoperative complications significantly higher in the case of surgeries performed by residents [8]. Our sample size was much smaller, but we only observed a difference between the groups in duration of surgery. In the learning curve period, it was 74.6 min in subgroup A1 (residents) and 64.13 min in subgroup B1 (consultants) (*p* < 0.05), while it was 57.3 min in subgroup A2 (residents) and 53.38 min in B2 (consultants) after the learning curve period (*p* < 0.05). In the two main groups, we compared the change in duration of surgery, the “dynamics” of learning: in Group A, duration of surgery decreased from 74.6 min to 57.3 min (*p* < 0.05), while in Group B, a drop from 64.13 min to 53.38 min was found (*p* < 0.05). It is interesting that the decrease in duration of surgery after the learning curve period was greater among residents. As they performed an increasing number of surgeries, they used the laparoscopic instruments with ever greater confidence, and both the surgeon performing the surgery and the surgical staff felt more confident in the laparoscopic situation [19, 20]. The more rapid improvement observed in the case of residents may be caused by the fact that, for many of them, laparoscopy was the primary surgical technique for appendectomy, as they had begun working in a period when the number of open appendectomies performed was small.

*Except for a few cases, we used Hem-o-lok clips for the closure of the appendix stump. Based on our experience, the time required to use Hem-o-lok clips is shorter than that involved in using Endoloops. It is also much easier for young surgeons with less experience, so we consider it a safer procedure. Endostaplers are easy to use, but their cost-effectiveness is low; on the other hand, Endoloops or endoscopic sutures represent a reliable, safe and cheap technique for closing the appendix base in the hands of an experienced laparoscopic surgeon. However, especially for residents in the learning curve period, it is a very challenging method, which can lengthen the operation time considerably. That is why we use Hem-o-lok clips as a gold standard for closing the stump of the appendix.*

*Use of a standardised technique described in a step-by-step manner can easily be learned by residents and may contribute to an improvement in outcomes [*21*]. This low-cost technique can also enable young residents to learn advanced laparoscopic skills in laparoscopic appendectomy, even in cases of complicated appendicitis.*

*Based on our experience, the algorithm for the safe introduction of laparoscopic appendectomy is the following:*

1. *Basic skills training: a two-week “Basic laparoscopic skills” course (with a training box and live animals) at the beginning of residency.*
2. *First assistance: assisting in laparoscopic procedures (appendectomy, cholecystectomy, laparoscopic hernia repair and laparoscopic hiatal hernia repair) to acquire the basic skills and learn the standardised technique.*
3. *Practising the standardised appendectomy technique during the learning curve period (first 20 cases) under the supervision of a consultant surgeon proficient in both laparoscopic and open techniques.*
4. *Starting appendectomies independently.*

This is another reason why learning this basic technique is so important—it is encountered by residents in large numbers, and it may be of great assistance during their training to prepare them for subsequent, more complex laparoscopic surgeries. In many countries, residents must participate in laparoscopic training first, with the basic surgery types practiced on simulators. According to some studies, this training decreases subsequent intraoperative complications [22, 23]. Others suggest that real procedures performed in the OR are required for the actual development of skills and for the resident to become a professional surgeon [24].

According to a US survey, a large proportion of residents feel that they did not perform a sufficient number of laparoscopic procedures during their residency and therefore do not feel secure when they have to perform surgery independently [25, 26]. As a result, in 2007–2008, the Accreditation Council of Graduate Medical Education increased the mandatory number of laparoscopic surgeries to be performed during residency training: from 25 to 60 for simpler, so-called basic procedures, and from 9 to 25 for more complex, advanced procedures [26].

With the spread of laparoscopy, increased attention must be paid to the training of residents, and there is a need to implement standardized training models, as it is clear that, in our case, laparoscopic appendectomy is a technique that can also be used safely by residents in the learning curve period—naturally under the supervision of a consultant. Learning this technique provides the residents with a valuable opportunity to perform more difficult, more complex laparoscopic surgeries with adequate safety in the future.

## Conclusions

Based on our experience laparoscopic appendectomy is a technique that can also be used safely by residents in the learning curve period as well. The rapid introduction of laparoscopy involves few risks, the surgery is also performed with sufficient safety by surgical residents. Comparing the resident and the consultant group based on patient demographics, intraoperative blood loss, conversion rate, hospital stay in days, and postoperative complications we did not find significant difference.

## Abbreviations

EAES: European Association of Endoscopic Surgery
LC: learning curve
NAS: negative appendectomy rank
